# Evolutionary Origin and Phylogeography of the Diploid Obligate Parthenogen *Artemia parthenogenetica* (Branchiopoda: Anostraca)

**DOI:** 10.1371/journal.pone.0011932

**Published:** 2010-08-04

**Authors:** Joaquín Muñoz, Africa Gómez, Andy J. Green, Jordi Figuerola, Francisco Amat, Ciro Rico

**Affiliations:** 1 Department of Wetland Ecology, Estación Biológica de Doñana (CSIC), Isla de la Cartuja, Seville, Spain; 2 Department of Biological Sciences, University of Hull, Hull, United Kingdom; 3 Instituto de Acuicultura de Torre de la Sal (Consejo Superior de Investigaciones Científicas), Ribera de Cabanes (Castellón), Spain; Field Museum of Natural History, United States of America

## Abstract

**Background:**

Understanding the evolutionary origin and the phylogeographic patterns of asexual taxa can shed light on the origin and maintenance of sexual reproduction. We assessed the geographic origin, genetic diversity, and phylogeographic history of obligate parthenogen diploid *Artemia parthenogenetica* populations, a widespread halophilic crustacean.

**Methodology/Principal Findings:**

We analysed a partial sequence of the Cytochrome *c* Oxidase Subunit I mitochondrial gene from an extensive set of localities (including Eurasia, Africa, and Australia), and examined their phylogeographic patterns and the phylogenetic relationships of diploid *A. parthenogenetica* and its closest sexual relatives. Populations displayed an extremely low level of mitochondrial genetic diversity, with one widespread haplotype shared by over 79% of individuals analysed. Phylogenetic and phylogeographic analyses indicated a multiple and recent evolutionary origin of diploid *A. parthenogenetica*, and strongly suggested that the geographic origin of parthenogenesis in *Artemia* was in Central Asia. Our results indicate that the maternal sexual ancestors of diploid *A. parthenogenetica* were an undescribed species from Kazakhstan and *A. urmiana*.

**Conclusions/Significance:**

We found evidence for multiple origin of parthenogenesis in Central Asia. Our results indicated that, shortly after its origin, diploid *A. parthenogenetica* populations underwent a rapid range expansion from Central Asia towards the Mediterranean region, and probably to the rest of its current geographic distribution. This contrasts with the restricted geographic distribution, strong genetic structure, and regional endemism of sexual *Artemia* lineages and other passively dispersed sexual continental aquatic invertebrates. We hypothesize that diploid parthenogens might have reached their current distribution in historical times, with a range expansion possibly facilitated by an increased availability of suitable habitat provided by anthropogenic activities, such as the spread of solar saltworks, aided by their natural dispersal vectors (i.e., waterbirds).

## Introduction

Parthenogenetic organisms tend to have broadly distributed genetic lineages, wider geographic distributions than their sexual relatives, and a propensity to occur in marginal areas (see [1] and references therein). Several non-exclusive hypotheses have been put forward to explain this phenomenon, ranging from their better colonisation abilities through reproductive assurance (i.e. a single female individual can reproduce and establish a new population) to their hybrid origin, polyploidy, or metapopulation dynamics (see [2] for a detailed discussion). Some of these hypotheses involve non-equilibrium scenarios for the advantage of parthenogenetic strategies (e.g. habitat dynamics or climatic trends could temporally favour parthenogenetic strategies). In this context, the use of phylogeographic tools to understand the historical dynamics of reproductive patterns might shed light on the widely debated maintenance of sexual reproduction [3]–[5].

Diapausing aquatic invertebrates represent an exceptional model to investigate the historical dynamics of sexual and parthenogenetic strategies. These organisms display an array of reproductive modes, often found in closely related taxa [6]. They produce resting eggs (i.e. encysted embryos in an arrested state of development), also called cysts, which form egg banks in the sediments of aquatic systems [7], facilitate survival of populations at extreme conditions [8]–[10], and allow dispersal via waterbirds (see [11] for a review). Nevertheless, despite their high potential for dispersal, most phylogeographic analyses in both sexual and cyclically parthenogenetic species show high levels of population genetic differentiation and regional genetic endemism [12]–[16]. In contrast, the few studies carried out in obligate parthenogenetic taxa indicate that genetic lineages are closely related, and identical or highly related clones are found over vast geographic areas [17], [18]. Examples of wide geographic distribution of genetic lineages have also been reported in a few species with mixed or alternative reproduction modes, including hermaphroditism, which, like obligate parthenogens, can potentially establish populations from a single propagule (i.e. diapausing egg) [15], [19], [20]. In all these cases, the historical availability of suitable habitat, either through human-mediated inoculations or postglacial processes, seems to have favoured organisms with unisexual modes of reproduction. Finally, these studies highlight that obligate parthenogenetic lineages are overwhelmingly very young from an evolutionary standpoint (but see [5]).

**Figure 1:**
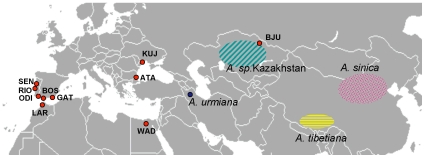
Geographic distribution of diploid *Artemia parthenogenetica* sampled sites, and geographic distribution of the Central Asian sexual species included in the genetic analyses (see text for details). Parthenogenetic population codes match those of Table 2. Due to the scale of the map, the parthenogen populations from Namibia and Australia are not shown.

Brine shrimps *Artemia* Leach, 1819 (Crustacea: Branchiopoda: Anostraca) inhabit hypersaline aquatic ecosystems worldwide, except Antarctica [21]. This genus comprises both sexual and obligately parthenogenetic lineages, which disperse passively through cysts. The binomen *Artemia parthenogenetica* Bowen and Sterling, 1978 (see [22] for taxonomic details) comprises an assemblage of parthenogenetic lineages with different levels of ploidy (from 2n to 5n). *Artemia parthenogenetica* is distributed over a vast geographic area, from the Canary Islands (in the west) to Australia (in the east) including large sections of Europe, Asia and Africa [23], [24], whereas its putative sexual relatives have more restricted distributions [21] (see Figure 1). The parthenogenetic diploid lineages are automictic (i.e. there is meiotic recombination during the cellular division but the products of meiosis fuse at some point to restore diploidy), while the polyploid lineages are apomictic parthenogens (i.e. offspring is genetically identical to the mother barring mutation) [25].

The closest sexual relative to parthenogenetic lineages of *Artemia* and the origin of parthenogenesis is debated [26], [27]. Nuclear sequence variation of the ribosomal Internal Transcribed Spacer 1 (ITS1) and mitochondrial DNA RFLP analysis of 16S ribosomal RNA indicated that the closest sexual relatives of confirmed diploid *A. parthenogenetica* (i.e. Namibia and Torre Colimena, in Italy [28]) were *A. urmiana* from Iran or *A. tibetiana* from Tibet [26]. On the other hand, some Chinese parthenogenetic lineages with an undetermined grade of ploidy were closely related to *A. sinica* from China. Although these analyses clarified the phylogenetic relationships of the genus, they did not provide sufficient resolution to shed light on the relationships between diploid *Artemia parthenogenetica* (*A. parthenogenetica* henceforth) lineages and their sexual relatives, nor on the phylogeographic history of their populations. Recent analyses based on sequence variation of the Cytochrome *c* Oxidase Subunit I (COI or cox1) mitochondrial gene have shown that the sexual Central Asian species, *A. tibetiana*, *A. urmiana*, and a sexual undescribed isolate from Kazakhstan [29] are closely related to each other and to *A. parthenogenetica* [16]. More detailed phylogeographic and phylogenetic analyses of these relationships are, however, required to resolve the origin and history of diploid parthenogenetic populations.

Here we examine the geographic distribution of mitochondrial lineages for *A. parthenogenetica* and investigate its phylogenetic relationships with the sexual Central Asian *Artemia* species. We assess the genetic diversity from a fragment of the COI mitochondrial gene of *A. parthenogenetica* populations from a large part of their distribution range, and include a new sexual population from the undescribed *Artemia* sp. from Kazakhstan. Our results shed light on the closest sexual relatives (i.e. the maternal evolutionary origin) and the geographic origin and history of parthenogenesis in *Artemia*, and indicate that the diploid parthenogens have undergone a very recent episode of geographic expansion.

## Results

### Genetic diversity and haplotype distribution

COI sequences (658 bp) from 102 individuals collapsed into a total of eight haplotypes from the 12 *A. parthenogenetica* populations analysed (see Table 1). Sequences did not show insertions, deletions or stop codons. The most commonly occurring haplotype was APD02 (found in 79% of individuals), which was found in all populations, except KUJ and WAD, in varying frequencies. All haplotypes except two (APD07 and APD08) differed from APD02 in just one or two substitutions. While nine individuals from ODI shared haplotype APD01, which differs by one substitution from the commonest haplotype, one individual from LAR and from ATA showed a third (APD03) and sixth (APD06) haplotype, respectively, which also differed from APD02 by a single substitution. Two haplotypes were exclusive to KUJ (APD04) and WAD (APD05), differing in one and two substitutions, respectively, from the common haplotype APD02. Additionally, the two shorter parthenogenetic *Artemia* sequences reported in GenBank from Australia (i.e. AY953368 and AY953369) differed in three and one substitutions, respectively, from the APD02 haplotype (see Table 1 for details). In contrast, haplotypes APD07 and APD08 were closely related to the available *A. urmiana* haplotype (three and two substitutions, respectively). In summary, haplotypes from parthenogenetic populations formed a common and widespread lineage of six highly similar haplotypes (eight if including the Australian ones) despite the large geographic distances that separate the populations analysed, while two of them form a ‘rare’ and ‘localised’ lineage from a single population (ATA). Excluding the Australian haplotypes, both *H* and *π* were very low, with values of 0.3349 (±0.059) and 0.00072 (±0.0007), respectively.

**Table 1:** Detailed information on diploid *Artemia parthenogenetica* haplotypes (Hap.) and their relative frequencies, number of individuals analysed (*n*) per population, and the Accession Number (Acc. Num.) from GenBank database.

	Diploid parthenogenetic populations													Polymorphic sites
Hap	GAT *n* = 7	ODI *n* = 13	BOS *n* = 10	RIO *n* = 12	SEN *n* = 12	LAR *n* = 10	MAR *n* = 7	KUJ *n* = 2	BJU *n* = 6	WAD *n* = 6	NAM *n* = 9	ATA n = 8	GenBank Acc. Num	11111112233444444555555566 157802345781334035689012345724 986532957817513631089280640439
APD01	—	0.69	—	—	—	—	—	—	—	—	—	—	DQ426824	TAGTTAATTTGACGCCCCTGAAGGTGATGG
APD02	1.00	0.31	1.00	1.00	1.00	0.90	1.00	—	1.00	—	1.00	0.63	DQ426825	.............................A
APD03	—	—	—	—	—	0.10	—	—	—	—	—	—	DQ426826	..............T..............A
APD04	—	—	—	—	—	—	—	1.00	—	—	—	—	GU591380	............T................A
APD05	—	—	—	—	—	—	—	—	—	1.00	—	—	GU591381	.........................A.A.A
APD06	—	—	—	—	—	—	—	—	—	—	—	0.13	GU591382	...................A.........A
APD07	—	—	—	—	—	—	—	—	—	—	—	0.13	GU591383	CG.CCG..CCTG.A..G.C.GC.AC....A
APD08	—	—	—	—	—	—	—	—	—	—	—	0.13	GU591384	CG.CC...CCTG.A..G.C.GC.AC....A
													*AY953368	-.....G...............A...G.—
													*AY953369	-.....................A..... —

	Sexual relative species													
	UNK1^2^ *n* = ?	UNK2 *n* = 14	UM^1^ *n* = ?											
KAZ^2^	1.00	—	—										†DQ119653	............................NA
KAZSEX01	—	0.07	—										**†**GU591385	........C..G.ATTG.C..C.......A
KAZSEX02	—	0.14	—										**†**GU591386	........C..G.AT.G.C..C.......A
KAZSEX03	—	0.14	—										**†**GU591387	..A....CC..G.AT.GTC..C.......A
KAZSEX04	—	0.21	—										**†**GU591388	........C..G.A..G.C..C.......A
KAZSEX05	—	0.43	—										**†**GU591389	..A....CC..G.AT.G.C..C.......A
*A. urmiana* 1	—	—	1.00										¶DQ119651	CG.CC...CCTG.A..G.C..C.A.....A

Information on sexual relatives, *A. urmiana* (¶) and *Artemia* sp. from Kazakhstan (†) haplotypes, is also included. Parthenogen population acronyms correspond to those described in Table 3 (CODE). UNK1: Unknown locality (Kazakhstan); UNK2: ARC 1039 (Kazakhstan); UM: Urmia Lake (Iran). Asterisks indicate shorter sequences from Australian parthenogens downloaded from GenBank, and the symbol ‘**—**’ indicates loss of information at that site.

1 From Hou et al. [29];

2 From Pilla & Beardmore [27].

**Figure 2:**
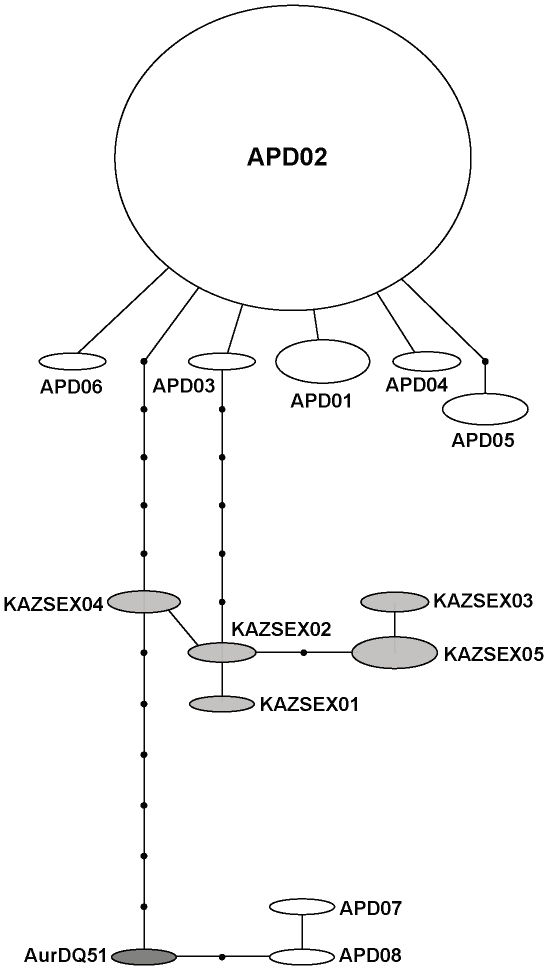
Statistical Parsimony network of all available diploid *Artemia parthenogenetica* haplotypes - in white - and two closely related Central Asian sexual species (*Artemia* sp. from Kazakhstan - in pale grey - and *A. urmiana* - in dark grey). Circle diameter is proportional to the relative haplotype frequency. The haplotype codes correspond to those listed in Table 1. Connecting lines indicate single substitutions and small black circles represent putative missing haplotypes. AurDQ51 =  *A. urmiana* haplotype with GenBank Acc Num DQ119651.

The haplotype network for diploid *A. parthenogenetica* haplotypes from the common lineage displayed a star shape (Figure 2) with APD02 as the central one. This haplotype showed a very wide geographic distribution, from Portugal (RIO, SEN populations) to Kazakhstan (BJU population), and Namibia (NAM population) (see Table 1 and Figure 2 for details). The haplotype from the sexual isolate species from Kazakhstan (collected in 1996) described by Hou et al. [30] did not differ from APD02. The rest of haplotypes from Kazakhstan (collected on 1988) differed by between six and ten substitutions from APD02. Specific locations for both samples from Kazakhstan are unknown, although their different haplotype composition suggests that they presumably come from different populations. *Artemia urmiana*, one of the closest recognized sexual species to *A. parthenogenetica*, showed 13–16 substitutions with respect to the commonest parthenogenetic haplotype APD02 (see Table 1 for details) but was closely related to two haplotypes from the ATA population. At the inter-specific level, average uncorrected COI sequence divergences (see Table 2) between the common parthenogenetic lineage and the two closest sexual relatives, *Artemia* sp. from Kazakhstan and *A. urmiana*, were 1.4% and 2.0%, respectively. A much higher value was found between parthenogens and *A. tibetiana* (5.2%).

**Table 2:** Net evolutionary divergence (i.e. the number of base differences per site from estimation of net average between groups of sequences) between *Artemia* species/lineages estimated by MEGA 4.0.

	*A. parthenogenetica*	*A*. sp. (DQ119653)	*A*. sp. (KAZSEX)	*A. urmiana*	*A. tibetiana*	*A. sinica*	*A. franciscana*
*A. parthenogenetica*	—	0.000	0.014	0.021	0.056	0.191	0.214
*A.* sp. (DQ119653)	0.000	—	0.014	0.021	0.057	0.192	0.215
*A.* sp. (KAZSEX)	0.014	0.014	—	0.009	0.048	0.196	0.204
*A. urmiana*	0.020	0.020	0.009	—	0.051	0.195	0.203
*A. tibetiana*	0.052	0.053	0.044	0.047	—	0.155	0.169
*A. sinica*	0.162	0.163	0.166	0.165	0.133	—	0.183
*A. franciscana*	0.181	0.182	0.173	0.173	0.146	0.159	—

Lower matrix *p*-distances, upper matrix corrected *K-2P* distances. *A*. sp.: undescribed sexual *Artemia*. DQ119653: Haplotype from the sexual isolate from Kazakhstan reported by Hou et al. [29]. KAZSEX: Haplotypes of sexual *Artemia* sp. from Kazakhstan obtained in this study (see Table 1).

### Mismatch distribution

**Figure 3:**
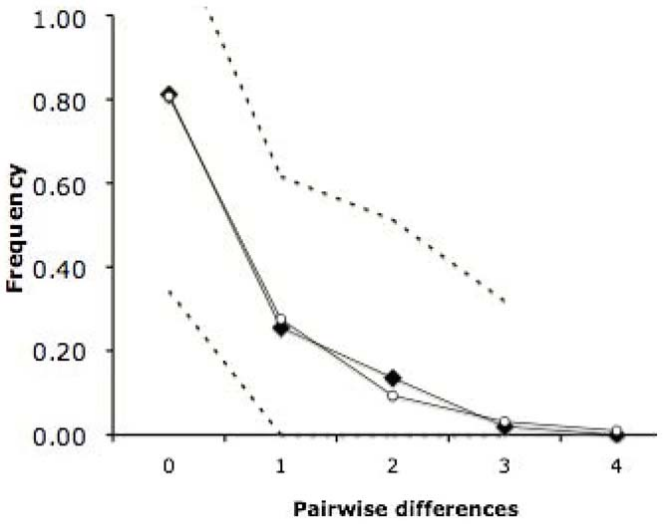
Mismatch distribution from the whole diploid *Artemia parthenogenetica* dataset. The solid black line with black squares indicates the observed distribution, and the solid black line with the white circle indicates the simulated distribution based on the sudden expansion model performed with Arlequin software. Dashed lines represent the upper and lower confidence intervals (α = 0.05) based on 1000 pseudo-replicates.

The results of the mismatch distribution (see Figure 3) showed that the common parthenogenetic lineage, excluding the shorter Australian haplotypes, was compatible with the ‘unimodal’ distribution model of a rapidly expanding population [31]. Estimated parameters from the sudden expansion model were: mismatch observed mean  = 0.473 and mismatch observed variance  = 0.557, and the test of goodness-of-fit of the SSD gave a value of 0.0012 with a non-significant *p*-value of 0.601.

### Phylogenetic relationships

**Figure 4:**
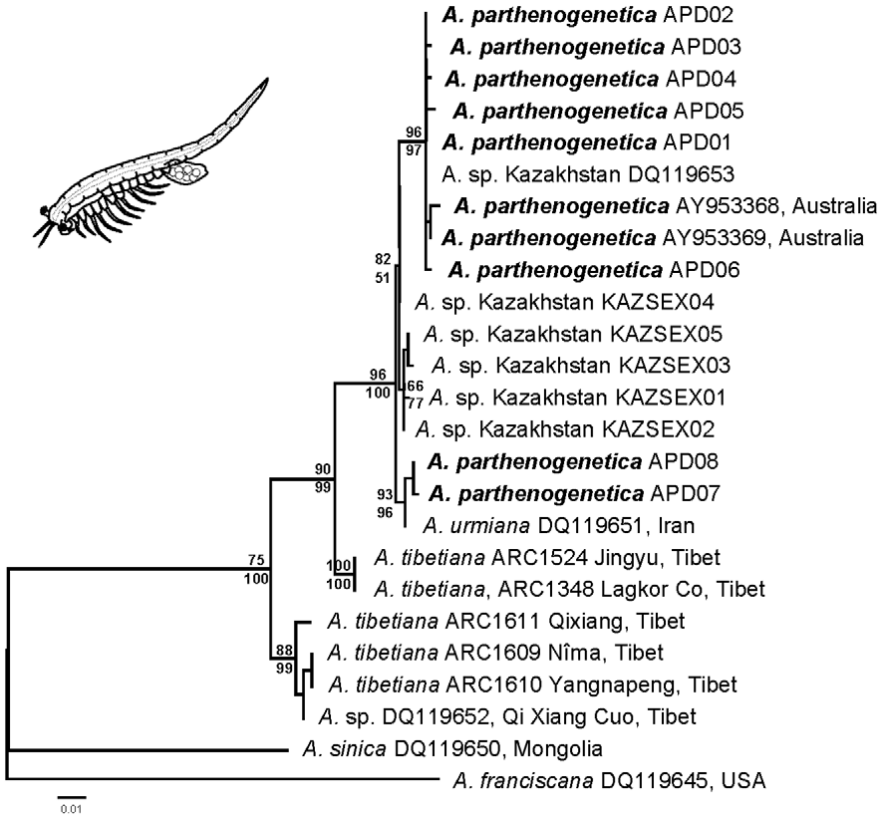
Phylogenetic relationships between diploid *Artemia parthenogenetica* (*A. parthenogenetica* in the tree) and its sexual relatives *A. urmiana*, *A. tibetiana*, and *Artemia* sp. from Kazakhstan using COI sequences. The topology inferred by Maximum Likelihood (ML) is shown. The Neighbor-Joining (NJ) tree showed a very similar topology. The percentage of replicate trees in which the associated taxa clustered together in a bootstrap test (1000 pseudo-replicates) is shown above (ML) and below (NJ) the branches. The sexual *A. franciscana* and *A. sinica* are used as outgroups. The schematic line drawing next to the ML tree represents a parthenogenetic *Artemia* female.

The phylogenetic reconstructions were robust to use of either NJ or ML, different outgroups or a range of different substitution models (results not shown). The phylogenetic tree (Figure 4) showed a similar topology in both NJ and ML analyses with a highly supported and closely related lineage containing most diploid parthenogenetic haplotypes and the haplotype from the sexual *Artemia* sp. from Kazakhstan. The rest of the sexual haplotypes from Kazakhstan and most of the parthenogenetic strains were closely related and formed a relatively well-supported group. The sister group to this branch contained *A. urmiana*, and two haplotypes from the diploid parthenogenetic Bulgarian population (ATA). *Artemia tibetiana* isolates formed a paraphyletic assemblage with respect to the above three lineages.

## Discussion

Our results show that diploid *Artemia parthenogenetica* has an extremely low level of mtDNA diversity across most of its known distribution range. The closest sexual relatives of this parthenogenetic lineage are found in Central Asia (Kazakhstan). A published sequence of a sexual strain from Kazakhstan [30] has a virtually identical COI haplotype to the commonest *A. parthenogenetica* one. We found another sexual population from Kazakhstan that was closely related to diploid parthenogens, and *A. urmiana* to be clearly more closely related to diploid parthenogens than *A. tibetiana*. A second rare parthenogenetic lineage was found in a single Bulgarian population and it was most closely related to *A. urmiana*.

### Geographic origin of parthenogenesis and putative ancestors

Although a previous hypothesis on the geographic origin of parthenogenesis in *Artemia*, based on allozyme data, proposed that the parthenogenetic lineage appeared in the Mediterranean Basin between 3 and 6 Myr ago [25], coinciding with the Messinian salinity crisis (around 6 My ago [32]), more recent data challenge this hypothesis. Baxevanis et al. [26] found different parthenogenetic *Artemia* lineages to be polyphyletic, with some of them more closely related to *A. sinica* and pointing out three other origins of parthenogenesis, all related to a group of Central Asian *Artemia* species (i.e. *A. urmiana*, *A. sinica*, and *A. tibetiana*). However, their analyses included both diploid and polyploid parthenogenetic *Artemia* isolates. In addition, the genetic variation found on nuclear and mtDNA markers (ITS1 and 16S) that they used was insufficient to discriminate between different hypotheses on the evolution of parthenogenesis. Therefore, they could not resolve the relationships between diploid parthenogenetic lineages and the group of sexual Central Asian species. Our present phylogenetic and phylogeographic analyses, which, unlike previous evolutionary studies on *Artemia*, include all known sexual taxa from Central Asia (i.e. *A. urmiana*, *A. tibetiana*, *A. sinica*, undescribed *Artemia* sp. from Kazakhstan, and *Artemia* sp. from Tibet), show that diploid *A. parthenogenetica* haplotypes from Eurasian, African and Australian populations are most closely related to two sexual species (i.e. *Artemia* sp. from Kazakhstan, and *A. urmiana*). Our results present more details on genetic relationships than previous studies [16], [26]. The close relationship between sexual *Artemia* sp. haplotypes from Kazakhstan and parthenogenetic haplotypes (see Table 1; see also Figure 3 and Figure 4) suggests that this undescribed sexual species is likely to be an ancestor to diploid *A. parthenogenetica* unrevealed to date. In addition, the close relationships between parthenogens, the sexual species from Kazakhstan and *A. urmiana* indicate, from a biogeographic perspective, that diploid *A. parthenogenetica* originated within a region of Central Asia around Iran and Kazakhstan. The lack of suitable calibrations precludes the use of molecular clocks in our study, but our findings suggest that the evolutionary origin of diploid parthenogenetic lineages is recent, probably within the Holocene. The diploid parthenogenetic lineages are usually involved in the origin of the polyploid parthenogenetic strains in animals [19], and this also appears to be the case in *Artemia* (the polyploid lineages appear to have arisen through autopolyploidy from the diploid ones [33]). Our results point to at least two recent origins of diploid parthenogenesis in *Artemia*, but when considering polyploid parthenogenetic lineages, parthenogenesis might have more than two independent origins (see [26]). Our dataset, however, is entirely consistent with a single origin of diploid parthenogenesis, which then spreads through introgression with different maternal lineages.

### Artemia parthenogenetica range expansion

Taken together, the extremely low genetic diversity, the close relationships between mtDNA haplotypes, and the results from mismatch distribution analysis for *A. parthenogenetica* indicate that this widespread anostracan underwent a rapid population expansion soon after its recent origin, likely associated with an expansion of suitable geographic range. If we assume a geographic origin in Central Asia, a process of rapid recent expansion from this area towards the Mediterranean region, and probably Australia, would explain the phylogeographic pattern observed on its populations. Overall, our results are similar to those reported in *Daphnia* species with obligate parthenogenetic reproduction mode, for which some young clonal lineages are very widely distributed [17], [18]. Indeed, the phylogeographic patterns of *A. parthenogenetica* starkly contrast with those for sexual anostracans [16], [34], whose populations are extremely subdivided, displaying a large degree of regional and local endemism. This is also apparent from the few samples of sexual Central Asian species included in this study, which show quite restricted geographic distributions (see Figure 4), but much higher diversity levels.

Given that both sexual and parthenogenetic anostracans have a similar potential to be dispersed via waterbirds [35], the contrasting phylogeographic scenario presented here could indicate that parthenogenesis provides a crucial advantage for a rapid spread of diapausing aquatic parthenogenetic organisms, particularly taking into account the low natural propagules pressures in these organisms [36]. Several studies suggest that, in passively dispersed aquatic invertebrates, parthenogens are indeed better and faster colonizers than their sexual relatives [17], [20], [36], [37]. The wide distribution of diploid *A. parthenogenetica* throughout Eurasia, Africa and Australia, the reduced genetic diversity found, and the evidence of a rapid population expansion, could be explained by a high colonization and establishment capacity after its origin, in comparison to their sexual relatives. This represents a similar pattern to the so-called ‘geographic parthenogenesis’ (i.e. distinct geographic distribution of parthenogenetic organisms and their sexual relatives; [38]) reported for other parthenogenetic species.

A colonisation advantage alone is not sufficient to explain the wide geographic distribution of parthenogenetic lineages, as habitat availability or a metapopulation structure conducive to frequent extinctions/recolonisations is also needed [2], [3]. In many cases, new habitats were made available for parthenogenetic lineages due to climatic changes (e.g. *Daphnia pulex*, [17]) or through human-mediated extra-range inoculations [18]. In the case of *Artemia parthenogenetica*, a pattern of natural postglacial colonisation could be a possibility. However, there is evidence that the sexual species *A. salina* survived glaciations in multiple Pleistocene refugia around the Mediterranean, suggesting that coastal hypersaline ponds persisted as available habitat throughout glacial maxima [16]. An alternative explanation could be that new suitable habitat for *A. parthenogenetica* was made available historically - at least since Phoenician times - through human activities to improve salt production in coastal wetlands. Indeed, one of the main habitats for *Artemia* are saltworks (i.e. artificial or natural saltpans managed by humans, at least for the last two millennia [39]). These habitats differ from natural hypersaline lakes in their carefully managed artificial salinity gradients in interconnected ponds, often fed from seawater, which creates high ecological similarities between them, as opposed to the widely different ionic composition and environmental regimes of natural *Artemia* habitats (see [40] and references therein). Although, under experimental conditions, diploid parthenogenetic *Artemia* are able to colonize habitats already occupied by the sexual sympatric *A. salina* and to displace them [41], [42], the ecological relationships between diploid *A. parthenogenetica* and their sexual relatives are poorly known. Indeed, differential spatial distribution suggestive of different ecological requirements of *A. parthenogenetica* and *A. urmiana* in and around Lake Urmia has been reported [43].

Although human management does not appear to have shaped the population structure in *A. salina* [16], our results are consistent with the role for humans in facilitating the spread of diploid *A. parthenogenetica* by creating new suitable coastal saltworks with rather homogeneous conditions. In this new setting, diploid *A. parthenogenetica* would have largely reached its current distribution assisted by natural dispersal through migratory waterbirds [24], [35]. On the other hand, recent artificial inoculation has been suggested as a possible way by which diploid parthenogens reached Australia [24] and, since it is thought that *Artemia* increases salt precipitation in salt works [39], a role for historical deliberate introduction cannot be ruled out. Finally, a more exhaustive sampling of *Artemia* within the vast and relatively unexplored Central Asian region would be desirable. The patchy and shallow knowledge of *Artemia* species (e.g. the unknown level of ploidy from many parthenogenetic strains not included in this study and the uncertainty on the distribution of *A. urmiana* [44]), and the difficulties in obtaining samples from Central Asia limit any large-scale study like ours. Our sampling design and results indicate that more precise inspection and research from Central Asian populations might lead to a better understanding of the details of the origin of parthenogenesis, population range expansion, and geographic speciation in *Artemia*, and would provide further information on evolutionary aspects of this passively dispersed aquatic invertebrate.

In conclusion, our results indicate that diploid *Artemia parthenogenetica* populations show very low mtDNA genetic diversity throughout most of their distribution range, with one widespread common haplotype and a few highly related haplotypes. Our phylogenetic and phylogeographic analyses suggest a very recent Central Asian origin of parthenogenesis in *Artemia*, with the closest sexual relatives being from Kazakhstan and Iran. Our findings also indicate a rapid range expansion of diploid parthenogenetic populations towards the Mediterranean region and probably to the rest of their current distribution.

## Materials and Methods

### Samples and study area

Samples from 12 parthenogenetic populations were collected from several Southern European countries, Central-East Asia (Ukraine and Kazakhstan), and Southern Africa (Namibia) (see Table 3 and Figure 2). All sampled sites were isolated hydrologically. In six sites, samples were collected in the field (adults or cysts), whilst at one site cysts were extracted from bird excreta collected ‘*in situ*’. All cysts were subsequently hatched and nauplii reared in the laboratory to adulthood to assess their reproductive mode through population sex ratio, using more than 2000 cysts per population (F. Amat, unpublished data; see also [28], [45]), and their ploidy using morphometric methods (for culture conditions and other details see [45], [46]). Populations were identified as diploid parthenogenetic following morphometric analyses, a reliable indicator of diploidy [28]. Adult specimens were then preserved in absolute ethanol until DNA extraction. The remaining samples (see Table 3) were obtained from the IATS (Instituto de Acuicultura Torre de la Sal, Castellón, Spain) cyst collection as dried cysts, which had been previously identified as diploid *A. parthenogenetica* (F. Amat, unpublished data). Previous cytogenetic analysis confirmed the diploid character (2n = 42, the same level of ploidy as the Asian sexual species) of the Namibia (NAM) population [47].

**Table 3:** Populations of diploid *Artemia parthenogenetica* analysed in the present study.

(CODE) Population	Country	Origin of samples	Coordinates
(GAT) Cabo de Gata saltpan	Spain	adults in saltpans	36.76N-02.22W
(ODI) Odiel saltpan		cysts in bird excreta	37.25N-06.99W
(BOS) El Bosque saltpan		adults in saltpans	36.79N-05.56W
(RIO) Rio Maior saltpan	Portugal	cysts in saltpans	39.36N-08.94W
(SEN) Senitra saltpan		cysts in saltpans	40.64N-08.67W
(LAR) Larache saltpan	Morocco	cysts in saltpans	35.20N-06.12W
(MAR) Margherita di Savoia saltpan	Italy	cysts in saltpans	41.38N-16.09E
(WAD) Wadi el Natrun	Egypt	IATS cyst collection	30.40N-30.32E
(KUJ) Kujalnicsky Liman	Ukraine	IATS cyst collection	46.72N-30.58E
(BJU) Bjurliv Lake	Kazakhstan	IATS cyst collection	51.75N-78.00E
(NAM) Vineta Swakopmund saltworks	Namibia	IATS cyst collection	22.67S-14.57E
(ATA) Atanasovko Lake*	Bulgaria	IATS cyst collection	42.57N-27.47E

*This population contained haplotypes closely related to *A. urmiana*, which were also included in the analyses.

IATS: Instituto de Acuicultura de Torre de la Sal (CSIC), Castellón-Spain.

In addition, one undescribed sexual *Artemia* population from Kazakhstan (Artemia Research Center code - ARC 1039, unknown locality) was analysed to increase the sampling of potential closest sexual relatives of *A. parthenogenetica*.

Published COI sequences from three sexual Central Asian species *Artemia* sp. KAZ, DQ119653; *A. urmiana*, DQ119651; and *A. tibetiana*, EF615584–90), and two parthenogenetic sequences from Australia (AY953368 and AY953369) were downloaded from GenBank. Sequences from *A. sinica* (DQ119650) and *A. franciscana* Kellogg, 1906 (DQ119645), the closest sexual relatives to sexual Central Asian species [16], [26], were used as outgroups in our phylogenetic analyses.

### DNA isolation, polymerase chain reaction, and sequencing

Total DNA was isolated from adult specimens using a modification of the CTAB protocol published by Palumbi [48] and Bossier et al. [49] with Proteinase K pre-treatment as well as RNAse post-treatment to remove the high amount of RNA observed in all samples. DNA from individual cysts was extracted using a HotSHOT protocol optimized for zooplanktonic diapausing eggs [50].

We used specific *Artemia* primers designed in the same position as primers LCO1490/HCO2198 [51] to amplify part of the COI mitochondrial gene (1/2COI_Fol-F: 5′-ATTCTACGAATCACAAGGATATTGG-3′, and 1/2COI_Fol-R: 5′-TACACTTCAGGATGGCCAAAAAATCA-3′; see [16] for details). PCR amplification was carried out under the following conditions: a cycle of 3 min at 94°C, followed by 35 cycles of 45 s at 94°C, 60 s at 45°C, and 60 s at 72°C, with a final step of 5 min at 72°C. PCR products were purified with MONTAGE-PCR columns (Millipore, Corp.) and sequenced in both directions using the BigDye Terminator Sequencing Ready Reaction v3.1 kit (Applied Biosystems), following manufacturer instructions, on an ABI 3130× l automated sequencer. Nucleotide sequences were edited by hand and aligned using Sequencher™ v4.5 software (Gene Codes Corp.). All polymorphic sites were manually rechecked. All new haplotypes found were deposited in GenBank (Accession Numbers DQ426824-DQ426826 and GU591380-GU591389; see Table 1).

### Data analyses

Standard diversity parameters (haplotype, *H*, and nucleotide, *π*, diversity), and mismatch distribution were calculated for the whole parthenogen dataset treated as a unique population (except the two shorter Australian haplotypes from GenBank and the two haplotypes from ATA related to *A. urmiana*), using Arlequin v. 2.0 [31]. Mismatch distribution analysis describes the distribution of pairwise nucleotide differences among haplotypes based on a model of sudden population expansion [52], [53]. The validity of the estimated mismatch parameters is tested using a Sum of Square deviations (SSD) test of goodness of fit, comparing observed and expected mismatch distributions. We calculated 95% confidence intervals with 1000 replicates using Arlequin's parametric bootstrap approach.

TCS ver. 1.21 [54] was used to reconstruct the genealogical relationships amongst haplotypes and detect any phylogeographic signature under Statistical Parsimony [55] with a confidential interval of 95%.

The evolutionary relationships of the parthenogens and their closest Central Asian sexual relatives were reconstructed using *A. sinica* and *A. franciscana* as outgroups. In order to include all available sequences, the COI haplotype alignment was trimmed to 478 base pairs (bp) for all sequences prior to phylogenetic analyses. We used two approaches. First, Neighbor-Joining (NJ) trees were reconstructed using evolutionary distances computed with the Maximum Composite Likelihood method in MEGA4.0 [56]. The robustness of the branches was assessed with 1000 bootstrap pseudo-replicates. MEGA 4.0 was also used to compute the net average sequence divergence between *Artemia* lineages. Secondly, we estimated the best-scoring Maximum Likelihood (ML) tree using a general time reversible model (GTR-GAMMA) of sequence evolution with 1000 bootstrap replicates computed by RAxML-VI-HPC v. 7.0.4 [57], [58] using the CIPRES portal at the San Diego Supercomputer Center (http://www.phylo.org).
